# Longitudinal analysis of immunocyte responses and inflammatory cytokine profiles in SFTSV-infected rhesus macaques

**DOI:** 10.3389/fimmu.2023.1143796

**Published:** 2023-03-22

**Authors:** Yi-Hui Li, Wen-Wu Huang, Wen-Qiang He, Xiao-Yan He, Xue-Hui Wang, Ya-Long Lin, Zu-Jiang Zhao, Yong-Tang Zheng, Wei Pang

**Affiliations:** ^1^ Key Laboratory of Animal Models and Human Disease Mechanisms of the Chinese Academy of Sciences, Kunming Institute of Zoology - The Chinese University of Hong Kong (KIZ-CUHK) Joint Laboratory of Bioresources and Molecular Research in Common Diseases, Center for Biosafety Mega-Science, Kunming Institute of Zoology, Chinese Academy of Sciences, Kunming, Yunnan, China; ^2^ Kunming College of Life Science, University of Chinese Academy of Sciences, Kunming, Yunnan, China; ^3^ Office of Science and Technology, The First Affiliated Hospital of Nanchang University, Nanchang, Jiangxi, China; ^4^ School of Life Sciences, University of Science and Technology of China, Hefei, Anhui, China

**Keywords:** SFTSV, non-human primates, rhesus macaques, inflammatory cytokines, immunocyte subsets

## Abstract

Severe fever with thrombocytopenia syndrome virus (SFTSV), an emerging bunyavirus, causes severe fever with thrombocytopenia syndrome (SFTS), with a high fatality rate of 20%–30%. At present, however, the pathogenesis of SFTSV remains largely unclear and no specific therapeutics or vaccines against its infection are currently available. Therefore, animal models that can faithfully recapitulate human disease are important to help understand and treat SFTSV infection. Here, we infected seven Chinese rhesus macaques (*Macaca mulatta*) with SFTSV. Virological and immunological changes were monitored over 28 days post-infection. Results showed that mild symptoms appeared in the macaques, including slight fever, thrombocytopenia, leukocytopenia, increased aspartate aminotransferase (AST) and creatine kinase (CK) in the blood. Viral replication was persistently detectable in lymphoid tissues and bone marrow even after viremia disappeared. Immunocyte detection showed that the number of T cells (mainly CD8^+^ T cells), B cells, natural killer (NK) cells, and monocytes decreased during infection. In detail, effector memory CD8^+^ T cells declined but showed increased activation, while both the number and activation of effector memory CD4^+^ T cells increased significantly. Furthermore, activated memory B cells decreased, while CD80^+^/CD86^+^ B cells and resting memory B cells (CD27^+^CD21^+^) increased significantly. Intermediate monocytes (CD14^+^CD16^+^) increased, while myeloid dendritic cells (mDCs) rather than plasmacytoid dendritic cells (pDCs) markedly declined during early infection. Cytokines, including interleukin-6 (IL-6), interferon-inducible protein-10 (IP-10), and macrophage inflammatory protein 1 (MCP-1), were substantially elevated in blood and were correlated with activated CD4^+^ T cells, B cells, CD16^+^CD56^+^ NK cells, CD14^+^CD16^+^ monocytes during infection. Thus, this study demonstrates that Chinese rhesus macaques infected with SFTSV resemble mild clinical symptoms of human SFTS and provides detailed virological and immunological parameters in macaques for understanding the pathogenesis of SFTSV infection.

## 1 Introduction

Severe fever with thrombocytopenia syndrome (SFTS) is an emerging tick-borne infectious disease caused by a novel phlebovirus (SFTS virus (SFTSV)) (1). Clinical manifestations of SFTS include fever, thrombocytopenia, leukocytopenia, bleeding tendency, and gastrointestinal symptoms, with a high mortality rate of 20%–30% (2). SFTSV poses an imminent threat to public health and is listed as one of the most dangerous pathogens by the World Health Organization (WHO) (3). The clinical symptoms of SFTS are non-specific, but commonly include fever, anorexia, myalgia, weakness, nausea, gastrointestinal symptoms, and regional lymphadenopathy, with hematological abnormalities of thrombocytopenia and leukocytopenia also frequently observed in laboratory tests (4, 5). Various risk factors are associated with fatal outcomes in SFTS, including older age, male, respiratory failure, hemorrhagic manifestations, disseminated intravascular coagulation (DIC), multiple organ dysfunction, hemophagocytic lymphohistiocytosis (HLH), and central nervous system symptoms (6, 7). Cytokine storm is also considered to be the main pathological feature of fatal SFTS in patients (8).

Animal models that can be infected with SFTSV and recapitulate features of SFTS are necessary to assess viral and immunological dynamics during infection, as well as to understand disease pathogenesis and evaluate vaccines and therapeutics. To date, non-lethal and lethal animal models of SFTSV have been established in mice, hamsters, rats, ferrets, and cats (9–16). Briefly, mild symptoms or slight pathological changes appeared in SFTSV infected adult wildtype mice, adult hamsters, adult rats and young adult ferrets (9, 11, 13, 14). Severe clinical manifestations or mortality appeared in SFTSV infected newborn wildtype mice, immunocompromised mice, and humanized mice, as well as newborn rats, aged ferrets, and cats (11–16). However, these animals are distinct from humans in terms of phylogenetics, physiology, anatomy, and immunology. In contrast, non-human primates are immunologically and physiologically similar to humans and present significant advantages over rodents and other species in several infectious disease models, such as acquired immune deficiency syndrome (AIDS) (17), coronavirus disease 2019 (COVID-19) (18), and tuberculosis (19) et al. Notably, a previous report has showed that rhesus macaques (*Macaca mulatta*) infected with SFTSV could faithfully exhibit mild clinical symptoms of SFTS in viremia, hematological, and biochemical parameters and in humoral immune responses, thus providing a suitable non-lethal model for studying the pathogenesis of SFTSV infection and evaluating immune responses against SFTSV (10).

To date, no specific vaccine or antiviral drug has been approved for the prevention or treatment of SFTSV infection, partly because the infection-induced antiviral immune response has not been fully described. Hence, it is necessary to dynamically detect changes in immunocyte subsets and the release of inflammatory cytokines during infection in a suitable animal model. Thus, in the current study, we assessed unexplored aspects of innate and adaptive immune responses and cytokine release induced by SFTSV infection in rhesus macaques.

## 2 Materials and methods

### 2.1 Virus and animals

The SFTSV of subtype E-JS-2013-24 (Genbank: KY362358.1 for segment M, and KY362310.1 for segment L) was provided by Professor Zhiwei Wu (Nanjing University, Nanjing, China) and propagated in Vero E6 cells (20). Healthy adult Chinese rhesus macaques (*Macaca mulatta*) (11-14 years old, *n* = 7, males) were enrolled in this study. All experimental procedures were performed in accordance with the guidelines approved by the Ethics Committee of the Kunming Institute of Zoology (approval number: IACUC-PE-2021-08-001).

### 2.2 Animals infection and samples collection

Animals were anesthetized by Zoletil and then received an inner thigh multiple hypodermic injection of 5 × 10^7^ TCID_50_ (50% tissue culture infection dose) of SFTSV in 1 mL of saline. Macaques were fed commercially prepared nonhuman primate food twice daily and monitored daily for abnormal appearance and behaviors. On days 0, 1, 2, 3, 4, 5, 6, 7, 9, 11, 14, 17, 21, 24 and 28 after inoculation, the animals were monitored for weights and temperatures. For blood sampling, 4 mL of venous blood was collected on days 0, 1, 2, 3, 4, 5, 6, 7, 9 and 11, and 8 mL of venous blood was collected on days 14, 17, 21, 24 and 28. Animal 1105155 was euthanized after 1 week of infection, 09431 was euthanized after 2 weeks of infection, 1103321 and 08067 were euthanized after 4 weeks of infection by overdose of pentobarbital sodium. Spleen, inguinal, axillary lymph node and bone marrow aspirates were obtained at the end points of experimental infections.

### 2.3 Virus detection in blood and tissues

Peripheral blood and bone marrow derived plasma virus RNA was extracted using the High Pure Viral RNA Kit (Roche, Germany) in accordance with the manufacturer’s protocols. Tissue RNA was extracted using RNAisoPlus Reagent (Takara, Japan) as previously described (21). For detection of SFTSV RNA, a THUNDERBIRD Probe One-Step qRT-PCR Kit (TOYOBO, Japan) was used in accordance with the manufacturer’s protocols. The primer set was as below: forward primer 5’-CAGTGCTACCCTGCAAAGAA-3’, reverse primer 5’-TGATGGCAAACATTAGCTTC-3’, and probe 5’-FAM-TCATCCTCCTTGGATATGCAGGCCT CA-BHQ1-3’.

### 2.4 Antibody titer detection

SFTSV glycoprotein (Gn) specific immunoglobulin M (IgM) and immunoglobulin G (IgG) antibodies were quantified by ELISA as described previously (22). Briefly, Gn (purchesed from Professor Zhiwei Wu) was coated onto ELISA plates at a concentration of 0.1 μg/mL at 4°C overnight. After washing 3 times, 100 μL blocking buffer with 5% albumin bovin V (A8020, Solarbio) in PBS was added to the plates and incubated at 37°C for 1 hour. After washing, 100 μL serially diluted control or experimental plasma was added and incubated at 37°C for 1 hour. Then the plates were washed and rabbit anti-monkey IgG-HRP (1:10,000 dilution, BS-0335R, Bioss) or rabbit anti-monkey IgM-HRP (1:10,000 dilution, BS-0336R, Bioss) was added respectively, and incubated at 37°C for another 2 hours. Subsequently, 3,3′,5,5′-tetramethylbenzidine (TMB, MilliporeSigma) substrate was added and incubated at 37°C for 15 minutes. Finally, 50 μL stop solution (C04-01003, Bioss) was added to stop the reaction and the OD values were measured at 450 nm using the 800TS (BioTec). The antibody titers of the plasma samples were determined as the last dilution present an OD value above 2-fold that of the average control values.

### 2.5 Immunofluorescence

Spleen and inguinal lymph node tissues fixed in 4% paraformaldehyde and immunofluorescence was performed as described previously (23). The antibodies used in this experiment included: Rabbit-anti-SFTSV HB29 (Abnova, USA), Goat Anti-Rabbit IgG H&L (Alexa Fluor^®^ 647) (Abcam, UK). Experimental results were observed on a Leica DMI4000B Microsystem (Leica Microsystems, Wetzlar, Germany).

### 2.6 Coagulation, hematological and biochemical parameters

Counts of white blood cells (WBCs), red blood cells (RBCs), platelets and levels of platelet crit (PCT), mean platelet volume (MPV) in blood samples were stored in ethylene diaminetetra acetic acid and measured using BC-2800 automated haematology analyser (Mindray^®^, China). The plasma levels of AST, ALT, creatine kinase, albumin, and blood urea nitrogen and serum creatinine were measured using a Dimension EXL 200 Integrated Chemistry System (Siemens Healthcare Diagnostics, Delaware, USA). Coagulation blood samples were collected with tubes containing 3.2% sodium citrate solution (9:1, v/v) and analyzed the thrombotic indexes (PT, APTT, TT, and FIB) by RAC-2880 automated blood coagulation analyzer (Qayto, China).

### 2.7 Flow cytometric analysis

Flow cytometric analysis was performed according to standard flow cytometric procedures, as described previously (24). The absolute number of CD4^+^ T, CD8^+^ T, B, NK and monocytes were stained with: anti-CD3-APC-Cy7 (clone SP34-2); anti-CD4-PerCP-Cy5.5 (clone RPAT4); anti-CD8a-PE-Cy7 (clone RPAT8); anti-NKG2A-PE (clone REA110); anti-CD14-APC (clone M5E2) and anti-CD20-FITC (clone H1). Immunocytes phenotyping was characterized with following antibodies: T lymphocytes were stained with anti-CD3-APC-Cy7 (clone SP34-2); anti-CD4-PE-Cy7 (clone RPAT4); anti-CD95-PE-Cy5 (clone DX2); anti-CD28-APC (clone 15E8); anti-HLA-DR-FITC (clone G46-6). B lymphocytes were stained with anti-CD20-APC-Cy7(clone 2H7); anti-CD27-PerCP-Cy5.5 (clone M-T271); anti-CD21- APC (clone B-ly4); anti-IgD-PE-Cy7 (clone AT-1); anti-CD86-PE (clone FUN-1); anti-CD80-FITC (clone B7-1). NK cells were stained with anti-CD3-APC-Cy7 (clone SP34-2); anti-NKG2A-PE (clone REA110); anti-CD8-PE-Cy7 (clone RPAT8); anti-CD16-FITC (clone 3G8); anti-CD56-PerCP-Cy5.5 (clone B159); anti-HLA-DR-APC (clone G46-6). Macrophage and dendritic cells were stained with anti-CD3-APC-Cy7 (clone SP34-2); anti-CD20-APC-Cy7 (clone 2H7); anti-CD14-PerCP-Cy5.5 (clone M5E2); anti-HLA-DR-APC (clone G46-6); anti-CD11c-PE-Cy7 (clone B-ly5); anti-CD123-PE (clone 7G3); anti-CD16-FITC (clone 3G8). All samples were tested on the BD FACSVerse flow cytometer (BD, USA). Data analyzed by FlowJo7.6 software.

### 2.8 Cytokine assays

Cytokines in plasma including IL-6, IL-10, CXCL10 (IP-10), IL-1β, IL-12p40, IL-17A and MCP-1 were analyzed using a LEGENDplex™ Non-Human Primate (NHP) Inflammation Panel (BioLegend, USA) according to the manufacturer’s instructions as described previously (25).

### 2.9 Statistical analysis

Baseline and follow-up data were compared using the paired t-test or Wilcoxon matched-pairs test, according to the distribution of the variables analyzed by Kolmogorov test. Pearson’s rank test was used to determine correlations between cytokine and immunocyte subsets during SFTSV infection. All data analyses were performed using GraphPad Prism 8 (GraphPad Software, USA). *P* < 0.05 was considered statistical significance.

## 3 Results

### 3.1 Detection of plasm SFTSV specific antibody and viral load in peripheral blood and other tissues

**Figure 1:**
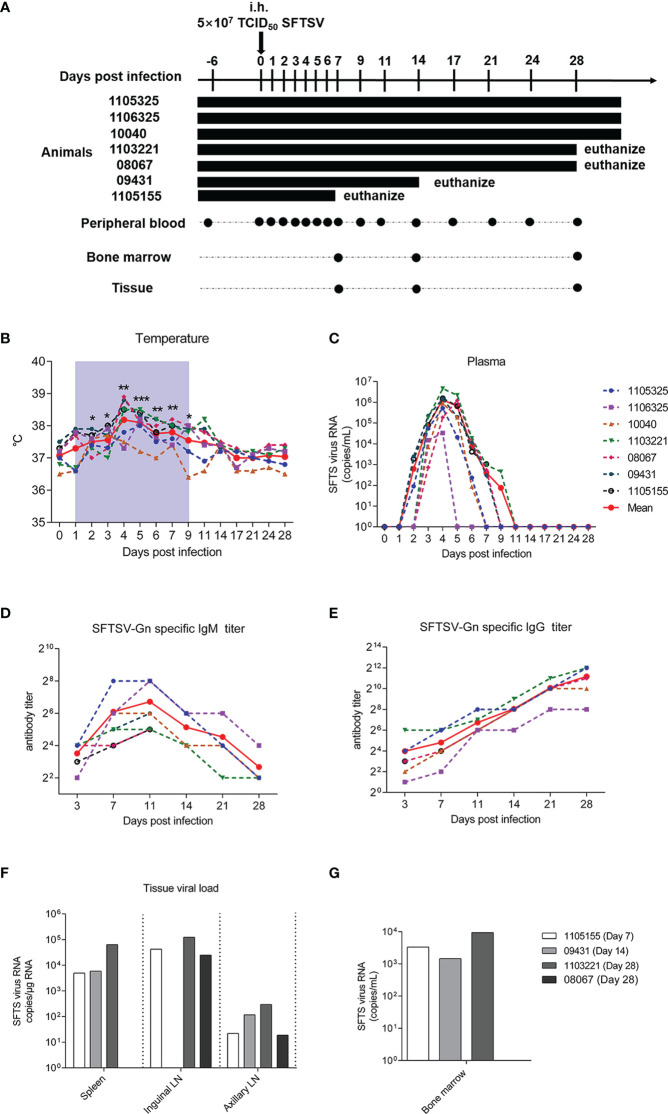
SFTSV detection in Chinese rhesus macaque plasma and tissue samples. **(A)** Outline of SFTSV challenge and tissue sampling. In total, 5 × 10^7^ TCID_50_ SFTSV (E-JS-2013-24 strain) was intramuscularly injected into seven rhesus macaques. Multiple tissue and plasma samples were collected at indicated time points. Macaque 1105155 was euthanized 1 week after inoculation, 09431 was euthanized 2 weeks after inoculation, and 1103321 and 09431 were euthanized 4 weeks after inoculation. Continuous temperature **(B)** and SFTSV RNA copy detection in plasma **(C)**. SFTSV-Gn specific IgG **(D)** and IgM **(E)** detection in plasma. SFTSV RNA copy detection in tissues **(F)** and bone marrow **(G)**. Gray-shaded areas indicate duration of viremia. Baseline (day 0) and follow-up data were compared using the paired t-test or Wilcoxon matched-pairs test, according to the distribution of the variables analyzed by Kolmogorov test. *P < 0.05, **P < 0.01, ***P < 0.001.

To study the pathogenesis, clinical symptoms, and host immune response under SFTSV infection, seven male Chinese rhesus macaques were subcutaneously injected with SFTSV (5 × 10^7^ TCID_50_). To examine viral replication and immune response in different tissues of infected macaques, one macaque was euthanized at 7 days post-infection (dpi), one macaque was euthanized at 14 dpi, two macaques were euthanized at 28 dpi, and three macaques were kept alive. Viral RNA copies were detected by real-time polymerase chain reaction (PCR) in peripheral plasma, spleen, inguinal lymph node, axillary lymph node, and bone marrow samples Peripheral blood samples were collected continuously, and tissue samples were obtained at each infection endpoint (**Figure 1A**). Compared with uninfected baseline (day 0), body temperature increased significantly in the infected macaques from day 2, peaked on day 4, and then decreased slightly but remained at a higher level than baseline to day 9 in the early acute infection. Thereafter, temperatures gradually declined to normal levels from days 11 to 28 (**Figure 1B**). These body temperature changes are similar with those observed in human patients (1), suggesting that the macaques were successfully infected with SFTSV. To confirm the infection in these macaques, plasma viral load and virus-specific antibody responses were detected. The plasma viral RNAs increased on day 1, peaked on day 4 (average of 1.5 × 10^6^ copies/mL), sharply declined and waned from day 5, and became undetectable by day 9 (**Figure 1C**), mimicking the viremia observed in human patients (12, 26). Virus-specific IgMs appeared on day 3 at a relatively low level of 1:8, and rose to 1:64 from days 7 to 15, thereafter they were gradually decreased (**Figure 1D**). Virus-specific IgGs appeared on day 3 at 1: 16, and increased constantly during the observing time of infection, reaching a high level of 1: 2,048 on day 28 (**Figure 1E**). Interestingly, although virions were rapidly eliminated in the blood, moderate levels of SFTSV RNA (ranging from 19 to 296 copies/mL) were consistently detected in the axillary lymph nodes of all infected macaques over the 28 days of infection. Consistently, high levels of SFTSV RNA were detected in the inguinal lymph nodes of 1105155 (day 7), 1103321 (day 28), and 08067 (day 28), ranging from 0.25 to 1.23 × 10^5^ copies/mL, and in the spleens of 1105155 (day 7), 09431 (day 14), and 1103321 (day 28), ranging from 0.5 to 6.4 × 10^4^ copies/mL (**Figure 1F**). Immunofluorescence also confirmed the presence of the SFTSV-Gn protein in the spleens and inguinal lymph nodes of macaque 08067 (28 dpi) (**Figure S1**). These results suggest that lymphoid tissues are the main sites of viral replication and potential reservoirs of SFTSV, even after viral clearance from plasma, consistent with observations in several human autopsy reports (14, 27, 28). Importantly, we found that SFTSV could enter the bone marrow early on day 7 (viral load of 3.3 × 10^3^ copies/mL) and virions were present from days 14 to 28 (1.5 and 9.4 × 10^3^ copies/mL, respectively), implying that SFTSV is constantly replicating at this tissue site (**Figure 1G**). As platelets are formed and differentiated in bone marrow, SFTSV can lead to thrombocytopenia, an interesting finding for further studies. Our results demonstrated that SFTSV is rapidly eliminated from the plasma of SFTSV-infected rhesus macaques but persists for a prolonged period in lymphoid tissues and bone marrow.

### 3.2 Changes in hematological and biochemical parameters and coagulation function after SFTSV challenge

**Figure 2:**
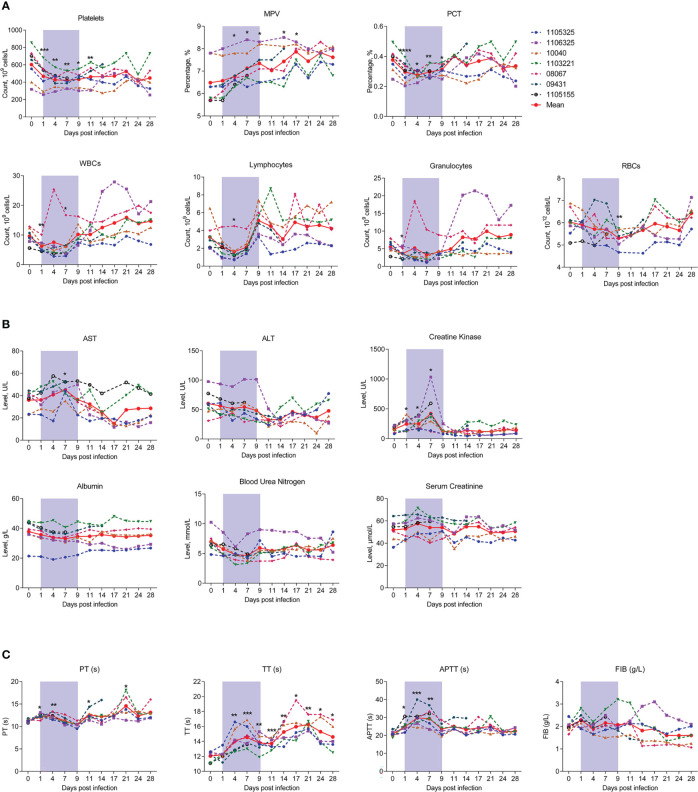
Kinetics of hematological, biochemical, and coagulation parameters in SFTSV-infected rhesus macaques. Hematological **(A)**, biochemical **(B)** and coagulation parameters **(C)** were detected in SFTSV-infected rhesus macaques. RBC, red blood cell; WBC, white blood cell; PCT, platelet crit; ALT, alanine aminotransferase; AST, aspartate aminotransferase; PT, prothrombin time; TT, thrombin time; APTT, activated partial thromboplastin time; FIB, fibrinogen. Gray-shaded areas indicate duration of viremia. Baseline (day 0) and follow-up data were compared using the paired t-test or Wilcoxon matched-pairs test, according to the distribution of the variables analyzed by Kolmogorov test. *P < 0.05, **P < 0.01, ***P < 0.001.

To determine whether SFTSV infection in macaques can mimic the clinical symptoms observed in humans, we measured hematological and biochemical parameters and coagulation function in the macaques before virus infection (day 0) and at 1, 4, 7, 9, 11, 14, 17, 21, 24, and 28 dpi. No severe symptoms or deaths were observed in the SFTSV-infected macaques. Hematological tests showed that average platelet count decreased sharply from days 1 to 4 and remained at a significantly low level thereafter, even at the end of observation on day 28 (448.2 ± 169.4), with levels about 25% lower than those at baseline (602.6 ± 176.3), as observed in human patients (7). Mean Platelet Volume (MPV) trended upward on day 4, and remained at a significantly high level thereafter, even at the end of observation on day 28. Plateletcrit (PCT) values also trended downward on day 1, with the lowest levels on day 4 (27% lower than baseline), but increased gradually thereafter and reached normal levels by day 11. These findings suggested that both morphological characteristics and numbers of individual platelets were impaired. Consistent with the decrease in platelets, white blood cell (WBC), lymphocyte, red blood cell (RBC), and granulocyte counts also decreased substantially on days 1 to 4. Furthermore, similar quantitative trends were observed, with a slight increase in WBCs, lymphocytes, and granulocytes at day 7 and higher levels than baseline at later check points (WBCs and granulocytes at day 14, lymphocytes at day 9), while RBCs increased from day 7 and reached normal levels at day 14 (**Figure 2A**). Thus, the numbers of major hemocytes are unanimously disordered by SFTSV during early acute infection.

We also monitored changes in peripheral blood biochemical parameters during infection. Aspartate aminotransferase (AST) and creatine kinase (CK) were significantly up-regulated on day 7 and decreased persistently thereafter, implying liver function injury from viral infection. Other biochemical parameters, such as alanine aminotransferase (ALT), albumin (ALB), blood urea nitrogen (BUN) and serum creatinine (CREA) did not change significantly during infection (**Figure 2B**).

Considering the sharp decrease in platelets and plateletcrit during early infection, we also measured the main parameters of coagulation function. Results showed that prothrombin time (PT) increased significantly from days 1 to 4, slightly decreased from days 7 and 9, and then increased thereafter. Thrombin time (TT) showed a prolonged upward trend up to day 28. Activated partial thromboplastin time (APTT) also increased from days 1 to 7 (**Figure 2C**). These findings are analogous to those observed in humans (26, 29), and confirmed that platelet function is substantially impaired with viral replication in peripheral blood. However, fibrinogen (FIB) levels exhibited no change during infection, implying that this protein is not involved in SFTSV pathogenicity (**Figure 2C**).

Our results revealed that SFTSV infection in rhesus macaques can cause mild symptoms of infection, analogous to those in humans, including decreased leukocyte counts, mild liver and kidney injury, and abnormal coagulation (26, 29).

### 3.3 SFTSV-induced changes in count reduction, subset alteration, and activation of T lymphocytes

**Figure 3:**
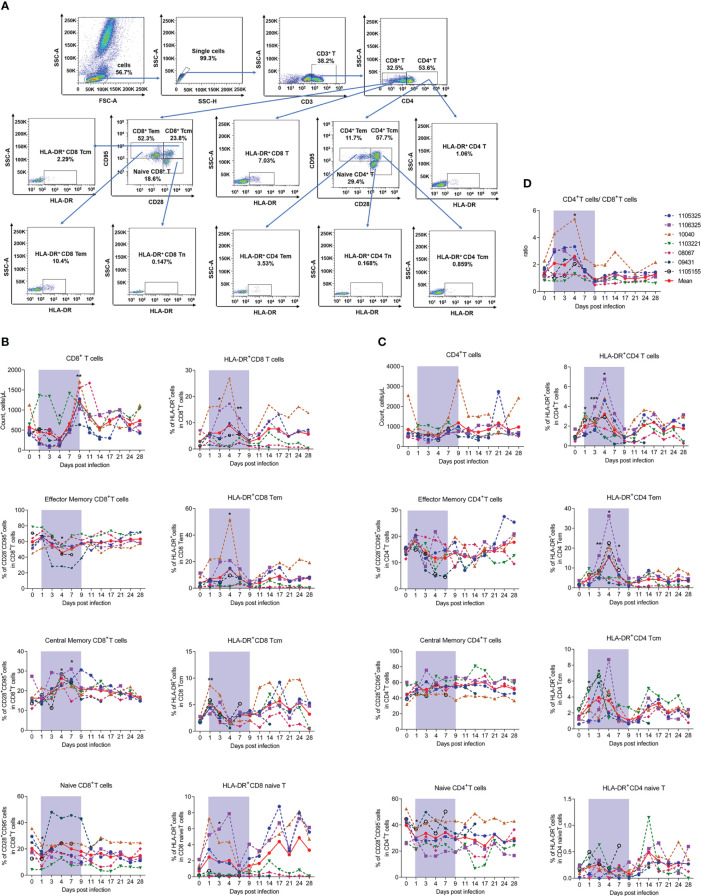
SFTSV-induced count reduction, subset alteration, and activation of T lymphocytes. **(A)** Gating strategy of flow cytometry for identification of T lymphocyte subpopulations. Counts, subsets, and activation (HLA-DR^+^) of CD4^+^ **(B)** and CD8^+^ **(C)** T cells were measured by flow cytometry. **(D)** Ratio of CD4^+^/CD8^+^ T cells. Tcm (central memory T cells, CD3^+^CD95^+^CD28^+^), Tem (effect memory T cells, CD3^+^CD95^+^CD28^-^), naive T (CD3^+^CD95^-^CD28^+^). Gray-shaded areas indicate duration of viremia. Baseline (day 0) and follow-up data were compared using the paired t-test or Wilcoxon matched-pairs test, according to the distribution of the variables analyzed by Kolmogorov test. *P < 0.05, **P < 0.01, ***P < 0.001.

To further investigate the host immune response during infection, we first detected changes in T lymphocyte number and subpopulation proportion in infected rhesus macaques. The gating strategies used to count CD4^+^ and CD8^+^ T lymphocytes and identify T lymphocyte activation and subpopulations are shown in **Figures S2**, **3A**, respectively. Results indicated that the number of CD8^+^ T cells decreased significantly by about 50% at 4 dpi (253.74 ± 178.58, *P* = 0.0427) compared to uninfected baseline (day 0, 574.37 ± 241.20), then increased sharply (**Figure 3B**), while the number of CD4^+^ T cells showed nonsignificant changes during infection (**Figure 3C**). Correspondingly, the CD4^+^/CD8^+^ T cell ratio increased significantly at 4 dpi (2.60 ± 1.31, *P* = 0.0148) compared to baseline (day 0, 1.38 ± 0.49) (**Figure 3D**).

Considering the ratio of CD4^-^CD8^-^ T cells to T cells was less than 5% and showed no significant changes during infection (**Figure S3**), and the limitation of our flow cytometry detection channels, we used the surface expression of CD3^+^CD4^-^ to label CD8^+^ T cells. Detection of CD8^+^ T lymphocyte subsets revealed that the decrease in CD8^+^ T cells was mainly due to CD28^-^CD95^+^ effector memory CD8^+^ T cells (CD8^+^ Tem) and CD28^+^CD95^-^ naive CD8^+^ T cells (CD8^+^ Tn). Notably, CD8^+^ Tem cells decreased by approximately 25% on day 4, while CD8^+^ Tn cells decreased by about 30% on day 1 from baseline but recovered rapidly by day 3. In contrast, CD28^+^CD95^+^ central memory CD8^+^ T cells (CD8^+^ Tcm) increased from baseline by approximately 50% from days 4 to 7 (**Figure 3B**). Considering the activation marker HLA-DR^+^, the percentage of HLA-DR^+^CD8^+^ T cells increased significantly from days 3 to 7, which may be due to the activation in CD8^+^ Tem cells, thus showing the same trend as CD8^+^ T cells. The percentage of HLA-DR^+^ in CD8^+^ Tcm and CD8^+^ Tn cells did not exhibit this pattern, instead increasing significantly on day 1 and decreasing thereafter (**Figure 3B**).

Interestingly, although the total number of CD4^+^ T lymphocytes showed limited variation during the viral infection process, subset detection showed that the percentage of CD28^-^CD95^+^ effector memory CD4^+^ T cells (CD4^+^ Tem) increased slightly, whereas CD28^+^CD95^-^ naive CD4^+^ T cells (CD4^+^ Tn) decreased on day 1. These cells also showed the opposite recovery trends during infection. The percentage of CD28^+^CD95^+^ central memory CD4^+^ T cells (CD4^+^ Tcm) showed no significant change during infection (**Figure 3C**), implying that naive CD4^+^ T cells differentiated into effector CD4^+^ T cells. Consistent with the changes in number, CD4^+^ Tem cells were the most activated among the three CD4^+^ T cell subtypes, with significant increases on days 3 (7.70 ± 3.77, *P* = 0.0041), 4 (15.32 ± 11.31, *P* = 0.0209), and 7 (6.36 ± 3.86, *P* = 0.0111) compared to baseline (day 0, 1.77 ± 0.97), while HLA-DR^+^CD4^+^ Tcm cells only increased slightly and CD4^+^ naive T cells exhibited no change during infection (**Figure 3C**). The down-regulation of CD4^+^ Tem cell number and activation suggests that these cells may be involved in viral infection.

Overall, the above results indicate that SFTSV infection in macaques can reduce the number of T cells (mainly CD8^+^ T cells) and can lead to alteration and activation of T cell subsets.

### 3.4 SFTSV-induced changes in count reduction, subset alteration, and abnormal class-switching of B cells

**Figure 4:**
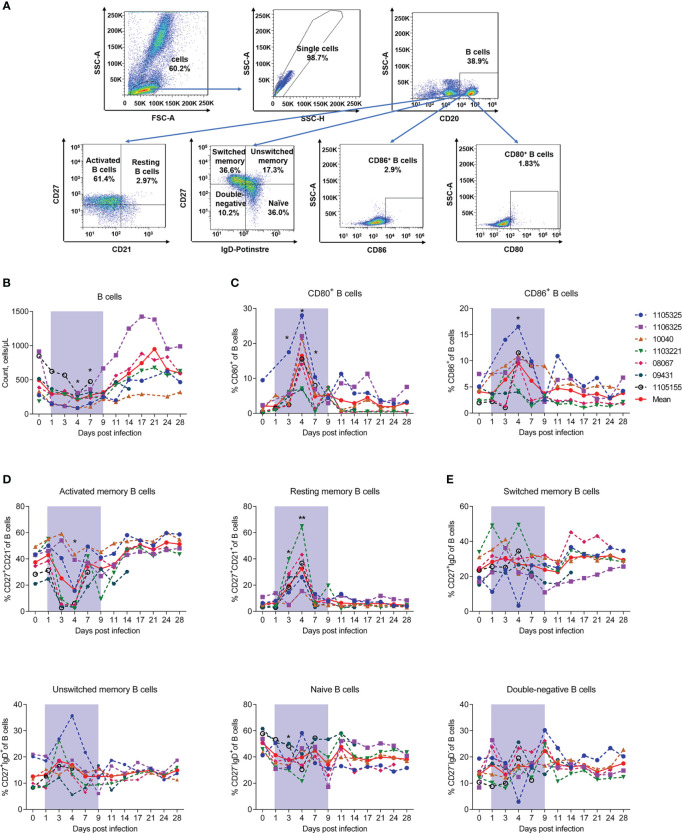
SFTSV-induced count reduction and activation, but abnormal class switching of B cells. **(A)** Gating strategy of flow cytometry for identification of B lymphocyte subpopulations. **(B)** B cell counting by flow cytometry. Percentage of CD27^-^CD21^+^ naive **(C)**, CD27^+^CD21^-^ activated **(D)**, and CD80^+^/CD86^+^ naive B cells. **(F)** Percentage of CD27^-^IgD^-^ double-negative, CD27^-^IgD^+^ naive, CD27^+^IgD^-^ switched memory, and CD27^+^IgD^+^ unswitched memory B cells. Gray-shaded areas indicate duration of viremia. Baseline (day 0) and follow-up data were compared using the paired t-test or Wilcoxon matched-pairs test, according to the distribution of the variables analyzed by Kolmogorov test. *P < 0.05, **P < 0.01.

We next measured the numbers and proportions of B lymphocytes. The gating strategies used to count and identify subpopulations of B lymphocytes are shown in **Figures S2**, **4A**, respectively. Results showed that the number of B cells significantly decreased by about 50% compared to baseline from days 4 and 7, then recovered and continued to increase from day 9 (**Figure 4B**). The co-stimulatory activation markers CD80^+^ and CD86^+^ were also evaluated and both showed a peak at day 4 (**Figure 4C**), the timepoint accompanied by the lowest number of B cells (**Figure 4B**). As reported in human patients, we divided B cells into resting (CD21^+^CD27^+^), activated memory B cells (CD21^−^CD27^+^), switched memory B cells (CD27^+^IgD^-^), unswitched memory B cells (CD27^+^IgD^+^), naive memory B cells (CD27^-^IgD^+^), and double-negative B cells (CD27^-^IgD^-^) and assessed the changes of these subsets during infection (20). Results showed that resting memory B cells increased on day 3 and peaked on day 4 (32.64% ± 16.18%, *P* = 0.0089) compared to baseline (day 0, 5.39% ± 2.52%). Activated memory B cells decreased significantly on day 4 (16.36% ± 16.39%, *P* = 0.0156) compared to baseline (day 0, 37.43% ± 9.21%) (**Figure 4D**). While the proportions of switched memory B cells, unswitched memory B cells, naive memory B cells, and double-negative B cells exhibited no obvious changes during infection (**Figure 4E**). Previous studies have reported that B cells are likely targets of SFTSV infection in humans (30, 31). Our results suggest that the decrease in B cells may be due to the down-regulation of activated memory B cells. However, whether this depletion is related to viral infection needs further exploration.

Collectively, these results indicate that SFTSV infection can induce a reduction in B cells and their activated subsets, but not B cell class switching dysfunction in the blood of rhesus macaques.

### 3.5 SFTSV-induced changes in frequency of monocyte, natural killer (NK) cell, and dendritic cell (DC) subsets

**Figure 5:**
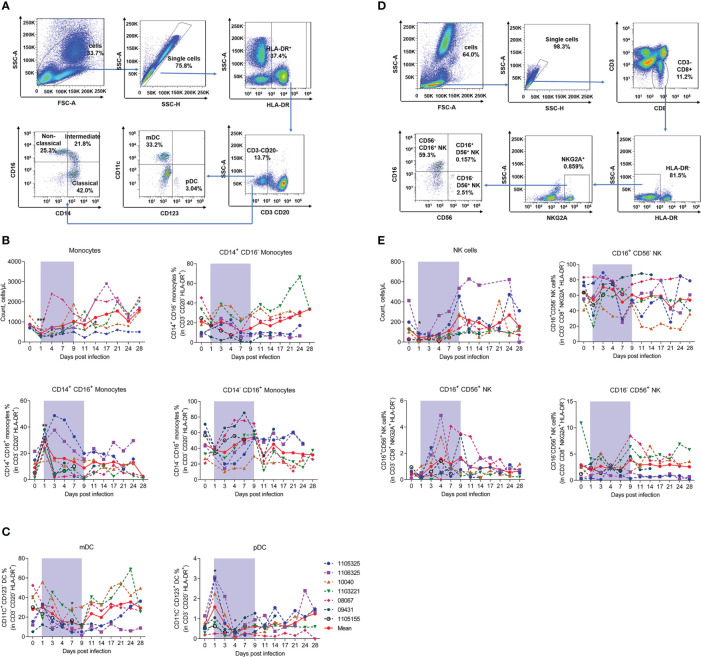
SFTSV challenge differentially impacted monocyte and DC subsets of PBMCs in rhesus macaque. **(A)** Gating strategy of flow cytometry for identification of monocytes and DCs. **(B)** Monocyte counting and percentage of CD14^+^CD16^+^, CD14^+^CD16^-^, and CD14^-^CD16^+^ subsets in CD3^-^CD20^-^HLA-DR^+^ cells. **(C)** Percentage of mDCs (CD3^-^CD20^-^HLA-DR^+^CD11c^+^CD123^-^) and pDCs (CD3^-^CD20^-^HLA-DR^+^CD11C^-^CD123^-^) in CD3^-^CD20^-^HLA-DR^+^ cells. **(D)** Gating strategy of flow cytometry for identification of NK cells. **(E)** NK counting and percentage of CD16^+^CD56^-^, CD14^+^CD56^+^, and CD16^-^CD56^+^ subsets in CD3^-^CD8^+^NKG2A^+^HLA-DR^-^ cells. Gray-shaded areas indicate duration of viremia. Baseline (day 0) and follow-up data were compared using the paired t-test or Wilcoxon matched-pairs test, according to the distribution of the variables analyzed by Kolmogorov test. *P < 0.05, **P < 0.01.

Next, we measured monocyte and DC numbers and subsets using the gating strategy shown in **Figure 5A**. Results showed that monocyte counts significantly decreased by about 50% on day 1 (414.29% ± 164.13%) and recovered on day 7 compared to baseline (871.43% ± 190.60%). Based on the CD14 and CD16 surface markers, monocytes can be divided into three subsets: i.e., classical (CD14^+^CD16^−^), non-classical (CD14^-^CD16^+^), and intermediate (CD14^+^CD16^+^) (32). Our results showed that the proportion of CD14^+^CD16^+^ monocytes was significantly up-regulated on day 1 (29.03% ± 6.29%) compared to uninfected baseline (9.11% ± 6.78%), but rapidly returned to baseline by day 3. No marked changes were observed in the CD14^-^CD16^+^ and CD14^+^CD16^-^ monocytes (**Figure 5B**). These results imply that CD14^+^CD16^+^ monocytes are the main evoked subset in viral infection. Our results also showed that the proportion of myeloid dendritic cells (mDCs) decreased continuously during infection, with the lowest level detected on day 7 (14.98% ± 8.83%) compared to baseline (27.92% ± 16.20%). The proportion of plasmacytoid dendritic cells (pDCs) increased significantly during early acute infection on day 1 (1.6% ± 1.1%), but decreased rapidly to normal levels on days 3–4, compared to baseline (0.6% ± 0.3%) (**Figure 5C**). We also measured the number and subsets of NK cells using the gating strategy shown in **Figure 5D**. NK cell counts decreased significantly on day 1 (43.20% ± 33.47%), but recovered to baseline levels on day 7 (130.97% ± 128.67%). During infection, albeit non-significantly, the proportions of CD16^+^CD56^+^ NK cells on day 4, day 7 and day 9 were 2.4-, 1.5- and 1.2-fold higher than that on day 0, respectively. While no changes were observed in the CD16^+^CD56^-^ and CD16^-^CD56^+^ subsets (**Figure 5E**).

These findings suggest that SFTSV infection reduces NK cells and monocytes but increases the proportion of CD14^+^CD16^+^ monocyte and CD16^+^CD56^+^ NK cell subsets, with distinct effects on pDCs and mDCs. The data of blood parameters, absolute counts of each leukocyte population and their specific subsets, together with their proportions, were summarized in **Supplementary Table 1**.

### 3.6 Abnormal cytokine profiles in infected rhesus macaques and correlation with immunocyte subsets

**Figure 6:**
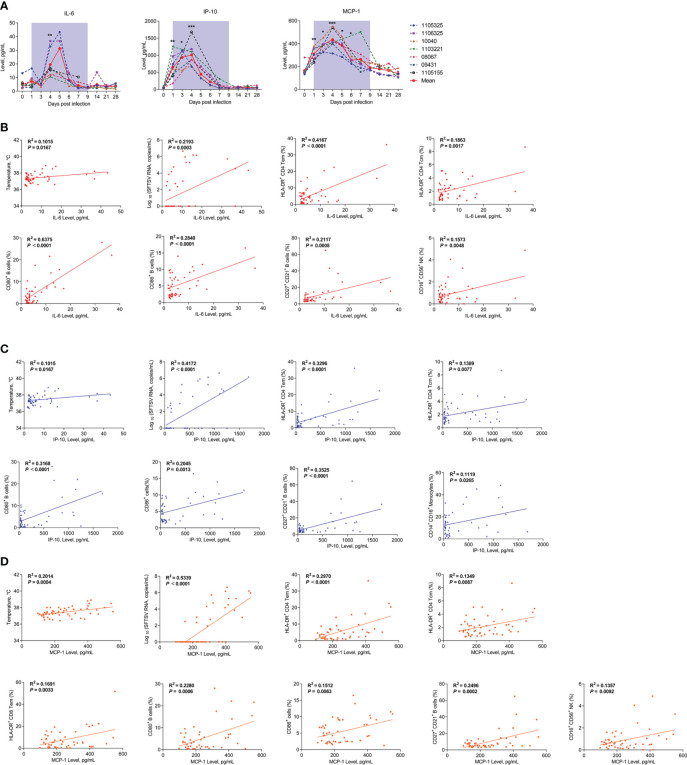
Kinetics of proinflammatory cytokines and correlations between cytokines and immunocytes in SFTSV-infected rhesus macaques. **(A)** Changes in plasma proinflammatory cytokines during SFTSV infection in rhesus macaques detected by LEGENDplex™ NHP Inflammation Panel. Pearson correlations of IL-6 **(B)**, IP-10 **(C)**, and MCP-1 **(D)** with activated T lymphocyte, B cell and monocyte subsets. Gray-shaded areas indicate duration of viremia. Baseline (day 0) and follow-up data were compared using the paired t-test or Wilcoxon matched-pairs test, according to the distribution of the variables analyzed by Kolmogorov test. Pearson’s rank test was used to determine correlations between cytokine and,viral load, body temperature and immunocyte subsets during SFTSV infection.*P < 0.05, **P < 0.01, ***P < 0.001.

Cytokine storm induced by SFTSV infection is a major pathological feature of patients with SFTS (8, 33). Therefore, clarifying the cytokine profiles and possible cellular sources of inflammatory factors in rhesus macaques is crucial for understanding the disease process. Here, we measured proinflammatory cytokine levels using a LEGENDplex™ Non-Human Primate (NHP) Inflammation Panel. Compared to baseline, interleukin-6 (IL-6) increased 5-fold on day 4, interferon-inducible protein-10 (IP-10) increased 14-fold on day 1 and 20-fold on days 3 and 4, and macrophage inflammatory protein 1 (MCP-1) increased 2.0–2.5-fold on day 1 but returned to normal by day 6 (**Figure 6A**). Levels of interleukin-10 (IL-10), interleukin-1 beta (IL-1β), interleukin-12 p40 (IL-12p40), and interleukin-17A (IL-17A) showed no significant changes (**Figure S4**).

Correlation analyses were conducted to investigate possible relationships between the cytokines and viral loads, body temperature, or leukocytes’ population in the SFTSV-infected macaques. The results showed that positive correlations existed between viral load, body temperature and the levels of cytokines, including IL-6 (**Figure 6B**), MCP-1 (**Figure 6C**), and IP-10 (**Figure 6D**). IL-6 is produced immediately by multiple innate immune cells and many other cells and plays a major role in removal of infectious agents by promoting differentiation of activated B cells into Ig-producing cells and regulating the direction of specific differentiation of T cells (34). The following correlation analysis showed that IL-6 levels were correlated with HLA-DR^+^CD4^+^ Tem and Tcm cells, CD80^+^, CD86^+^, and CD27^+^CD21^+^ B cells, and CD16^+^CD56^+^ NK cells (**Figure 6B**). MCP-1 is mainly secreted by monocyte/macrophages and can regulate the migration and infiltration of monocytes, memory T lymphocytes, and natural killer (NK) cells (35). The results showed that MCP-1 levels were correlated with HLA-DR^+^CD4^+^ Tem and Tcm cells, HLA-DR^+^CD8^+^ Tem cells, CD80^+^, CD86^+^, and CD27^+^CD21^+^ B cells, and CD16^+^CD56^+^ NK cells (**Figure 6C**). IP-10 is widely produced by various cell types, including monocytes, T lymphocytes, natural killer (NK) cells, endothelial cells, and stromal cells, and it can suppress the functions of T cells and NK cells (36). The results showed that IP-10 levels were correlated with HLA-DR^+^CD4^+^ Tem and Tcm cells, CD80^+^, CD86^+^, and CD27^+^CD21^+^ B cells, and CD14^+^CD16^+^ monocytes (**Figure 6D**). Together, these results suggest that the SFTSV infection in macaques are capable of inducing higher levels of IL-6, IP-10 and MCP-1; meanwhile, activated T and B cells and increased CD16^+^CD56^+^ NK cells and CD14^+^CD16^+^ monocytes may play important roles in cytokine secretion or interaction with the increased cytokines.

## 4 Discussion

SFTSV is a novel human pathogen originally classified as a virus genus but later designated as *Dabie bandavirus* (genus *Bandavirus*; family *Phenuiviridae*) by the International Committee on Taxonomy of Viruses in 2020 (37). SFTSV is primarily transmitted to humans through tick bites but can also be transmitted from animals *via* direct contact with blood or mucus (38–41). The development of adequate SFTSV-infected animal models is important to understand the pathogenesis of this pathogen. Non-human primates are regarded as gold standard animal models for studying the pathogenesis of some human infectious diseases (42). A previous study reported that cynomolgus macaques are not susceptible to SFTSV, exhibiting no overt clinical signs, no detectable virus in the blood, and no histopathological changes (43). However, young rhesus macaques (4-5 years old) can be infected with SFTSV and develop mild SFTS symptoms (10). Here, we observed similar symptoms and physical signs, including mild fever, leukopenia, thrombocytopenia, increased levels of serum AST, CK, and CREA, and delayed PT, TT, and APTT, present in middle-aged rhesus macaques (11-14 years old), confirming that SFTSV infection in rhesus macaques is similar to mild SFTS in humans. Furthermore, although virions in peripheral blood were rapidly eliminated by 9 dpi, virions in the bone marrow, spleen, and lymph glands persisted in the infected macaques for at least 28 dpi.

Many clinical studies have been performed on lymphocyte subpopulations and activation in SFTSV-infected patients, especially between patients during the acute infection and convalescent phases or showing mild and severe symptoms (20, 44–50). However, the exact timepoint of infection in these studies is unclear, and changes in peripheral blood lymphocytes and cytokines are usually evaluated days after disease onset (45, 46, 48–50). In humans, the incubation period of SFTSV is 7–14 days (51), followed by the onset of fever or illness. Therefore, studies in humans provide incomplete data regarding the dynamic changes in lymphocytes or cytokines during SFTSV infection. As such, macaque models may provide valuable virological and immunological information on the early stages of infection. In our SFTSV-infected macaques, we found that infection modulated the relative frequencies of several immune cell subsets. Unlike the marked decrease in CD4^+^ T cell counts and limited change in CD8^+^ T cell counts in human patients (45, 47), we found that CD8^+^ T cells diminished significantly during infection. The differences in infection characteristics observed between macaques and humans may be caused by various factors, including inherent differences in genetic background, basis difference, analysis of different infection stages, and different virus strains, infection routes and exposure doses (10); as well as the extrinsic differences in checking points during infection. As to the activation changes of CD8^+^ T cells, HLA-DR is an MHC cell receptor highly present on APCs and also expression on activated T cells (52). It appears in the late-stage activation of T cells and can serve as a marker of activated T cells (53). HLA-DR^+^ T cells are thought of highly proliferative and short-lived (54) and HLA-DR^+^ Tregs subset is described to be more cytotoxic (55). Report revealed that HLA-DR^+^ T cells are elevated in SFTSV-infected patients (53). Similarly, we found that HLA-DR^+^CD8^+^ T cells (including CD8^+^ Tem, CD8^+^ Tcm, and CD8^+^ naive T cells) and CD4^+^ T cells (especially CD4^+^ Tem and CD4^+^ Tcm) were significantly increased in the macaques during infection.

Previous studies have found that B cells are permissive to SFTSV infection (31, 50, 56). However, the effects of SFTSV on B cells in human patients remains controversial (45, 47, 57). Our results showed that B cells decreased in the macaques during infection, while B cell activation markers CD80^+^ and CD86^+^ (58) increased significantly, consistent with T cell activation. Based on CD21 and CD27 surface expression, we categorized B cells into resting (CD21^+^CD27^+^) and activated memory B cells (CD21^−^CD27^+^) (59). Our results showed that SFTSV infection increased the proportion of resting memory B cells and decreased the proportion of activated memory B cells in macaques. We also divided B cells into four subsets based on CD27 and IgD staining, as reported previously (60), i.e., switched memory B cells (CD27^+^IgD^-^), unswitched memory B cells (CD27^+^IgD^+^), naive memory B cells (CD27^-^IgD^+^), and double-negative B cells (CD27^-^IgD^-^). However, no major changes were observed in these subsets, similar to that reported in non-lethal SFTS patients (20). It has been reported that SFTSV infection can lead to the disorder of B cell development and maturation in patients. Although the proportion of CD80^+^CD86^+^ B cells increased, class switching failure appeared in SFTSV infection patients (20). This may because the virus can directly infect B cells, especially activated B cells which are considered as target cells of virus infection. The infected B cells may hinder the development and maturation of B cells (50). In the present study, the proportion of CD80^+^ and CD86^+^ B cells increased in the macaques, consistent with that observed in humans; while activated memory B cells and the number of B cells decreased, the underlying mechanism necessitates further exploration.

NK cells kill infected cells by secreting perforin and granzyme and play an important role in antiviral responses (61). In the SFTSV-challenged macaques, the absolute number of NK cells decreased in the early stage of infection, consistent with reports in humans (57, 62). Furthermore, we found that circulating CD56^+^CD16^+^ NK cells, major producers of cytotoxic cytokines (63), increased during the acute phase of SFTSV infection in macaques, suggesting they may play a major role in viral clearance.

DC-specific intercellular adhesion molecule 3-grabbing nonintegrin (DC-SIGN)-expressing DCs and monocytes are reported to be permissive to SFTSV infection (64, 65). Here, we found that the absolute number of monocytes was reduced in macaques during the early stage of infection. Previous single-cell sequencing of blood samples from SFTSV patients revealed a phenotypic shift in monocyte populations from classical (CD14^+^CD16^−^) to intermediate (CD14^+^CD16^+^) following SFTSV infection (20). High levels of antigen presentation-related molecules and inflammatory cytokines expressed by intermediate monocytes play an important role in rapid defense against pathogens (66). Consistently, we found that the frequency of intermediate monocytes (CD14^+^CD16^+^) increased during infection. Furthermore, the proportion of mDCs decreased during infection, whereas pDCs increased significantly during acute early infection and then decreased rapidly on days 3-4. As SFTSV entry in certain cell lines depends on the cellular receptor DC-SIGN (64), which is expressed in CD11c^+^ mDCs, but not pDCs (67), we inferred that mDCs rather than pDCs may be the target cells of SFTSV. In addition, pDCs are a specialized DC population with great potential to produce large amounts of type I interferon (IFN-1) and are involved in the initiation of antiviral immune responses (68), and therefore may play an important role in rapid defense against SFTSV.

Cytokine storm is considered a major pathological feature in patients with fatal SFTS (8, 33, 69). Liu et al. found that IFN-α, IFN-γ, G-CSF, MIP-1α, IL-6, and IP-10 concentrations are significantly elevated in patients with severe SFTS compared to patients with mild SFTS (69). He et al. also found that serum levels of IL-6, IL-10, IP-10, and MCP-1 can reflect disease severity (33). Here, in the mild SFTSV-infected macaques, the levels of IL-6, MCP-1, and IP-10 were significantly increased during infection. Correlation analysis also indicated that higher numbers of the virus were capable of inducing higher levels of IL-6, IP-10 and MCP-1. Meanwhile, the change of immunocyte subsets, which may be the main source of increased cytokines, and play an important role in regulating the release of inflammatory cytokines.

**Figure 7:**
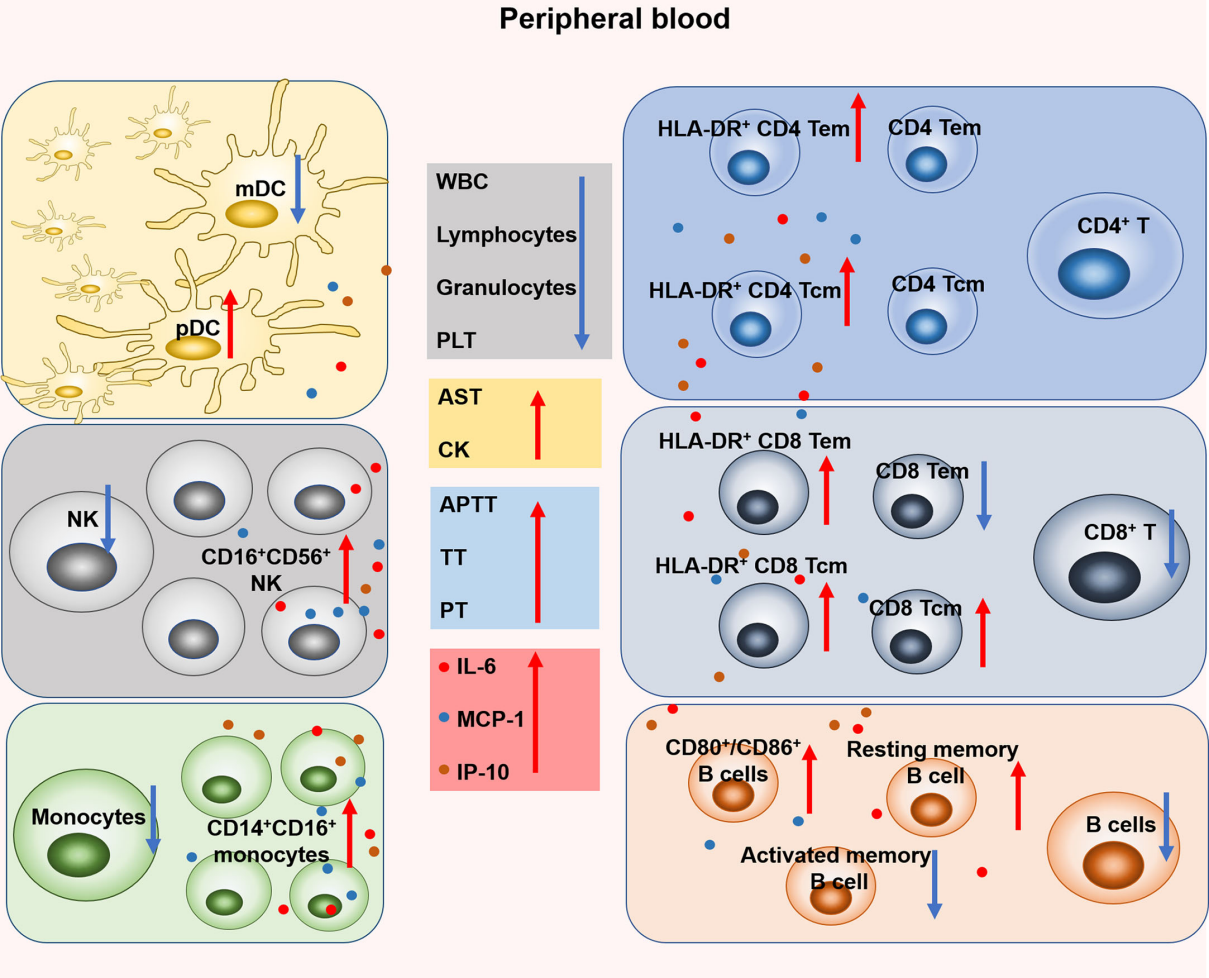
Changes of coagulation, hematology, biochemistry and leukocyte subsets in rhesus monkeys during the early stage of SFTSV infection. Specifically, the proportion of mDC decreased on days 4-9 and pDC increased on day 1. The number of NK cell decreased on days 1-3 and the proportion of CD16^+^ CD56^+^ NK increased on days 4-9. The number of monocytes decreased and the proportion of CD14^+^CD16^+^ monocytes increased on day 1. No significant change in the number of CD4^+^ T and the proportion of CD4 Tem, CD4 Tcm, but both the proportions of HLA-DR^+^ CD4 Tem and HLA-DR^+^ CD4 Tcm increased on days 3-7 and days 1-4, respectively. The number of CD8^+^ T decreased on day 4, and the proportion of CD8 Tem decreased on day 4. However, the proportion of CD8 Tcm increased on days 4-7. In addition, both the proportions of HLA-DR^+^ CD8 Tem and HLA-DR^+^ CD8 Tcm increased on day 1 and day 4, respectively. The number of B cells and the proportion of activated memory B cells decreased on days 4-7 and days 3, 4 respectively. The proportion of CD80^+^ and CD86^+^ B cells increased on days 3-7 and days 3, 4, respectively.

As shown in **Figure 7**, our study demonstrated the dynamic changes of coagulation, hematology, biochemistry and immunocyte subsets during the early stage of SFTSV infection and explored the correlation between changes in immunocyte subsets and inflammatory cytokines. The immune response detected in this mild infection model is similar to that observed in humans. These findings should help to elucidate the complex pathogenesis of SFTSV infection.

## Data availability statement

The original contributions presented in the study are included in the article/**Supplementary Material**. Further inquiries can be directed to the corresponding authors.

## Ethics statement

The animal study was reviewed and approved by the Ethics Committee of the Kunming Institute of Zoology.

## Author contributions

Y-TZ, and WP conceived and designed the experiments. Y-HL, W-WH, W-QH, X-HW, Y-LL, X-YH, and Z-JZ performed the experiments. Y-HL and W-WH performed data analyses. Y-HL, W-WH, WP, and Y-TZ wrote the manuscript. All authors contributed to the article and approved the submitted version.
