# Supplementary material for: Longitudinal analysis of immunocyte responses and inflammatory cytokine profiles in SFTSV-infected rhesus macaques
Source: Front Immunol. 2023 Mar 22;14:1143796. doi: 10.3389/fimmu.2023.1143796 (PMC10073517; doi:10.3389/fimmu.2023.1143796)
Supplement: Supplementary file 1 [file DataSheet_1.docx]

Supplementary Material

**Longitudinal analysis of immunocyte responses and inflammatory cytokine profiles in SFTSV-infected rhesus macaques**

Yi-Hui Li, Wen-Wu Huang, Wen-Qiang He, Xiao-Yan He, Xue-Hui Wang, Ya-Long Lin, Zu-Jiang Zhao, Yong-Tang Zheng, Wei Pang

*Correspondence: Yong-Tang Zheng, [zhengyt@mail.kiz.ac.cn](mailto:zhengyt@mail.kiz.ac.cn); or Wei Pang, [pangw@mail.kiz.ac.cn](mailto:pangw@mail.kiz.ac.cn).

**
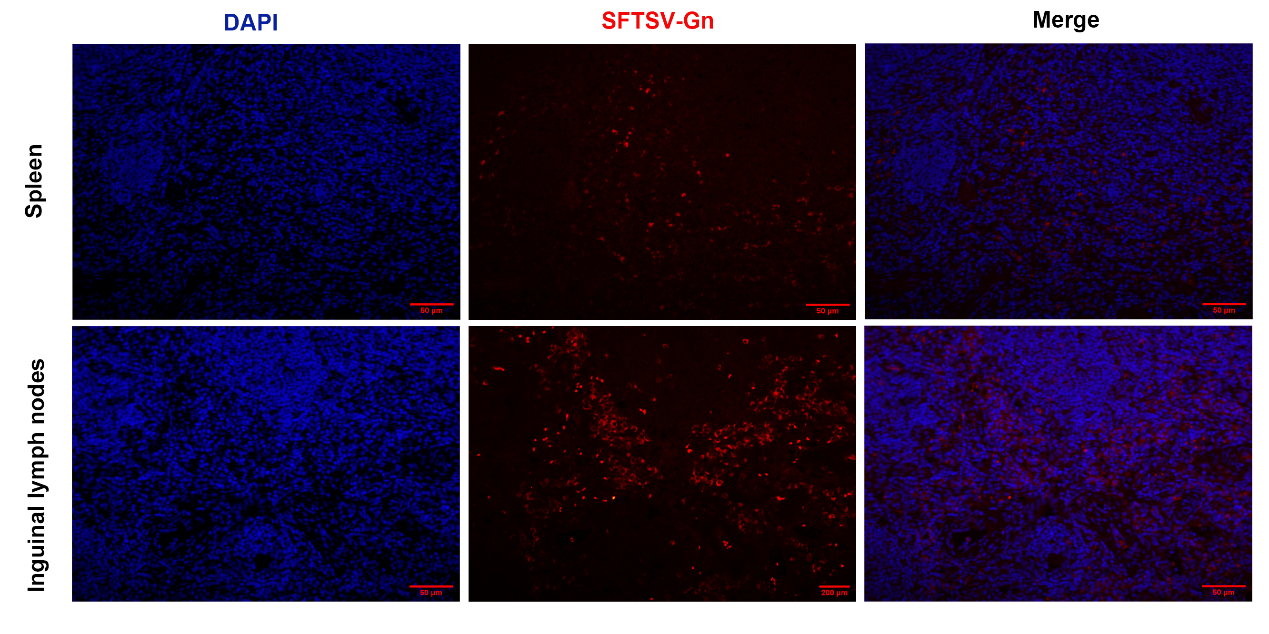
**

**Fig. S1.** SFTSV Gn protein was detected in inguinal lymph glands and spleen of macaque 08067 (28 dpi) by immunofluorescence (original magnification × 200).

**
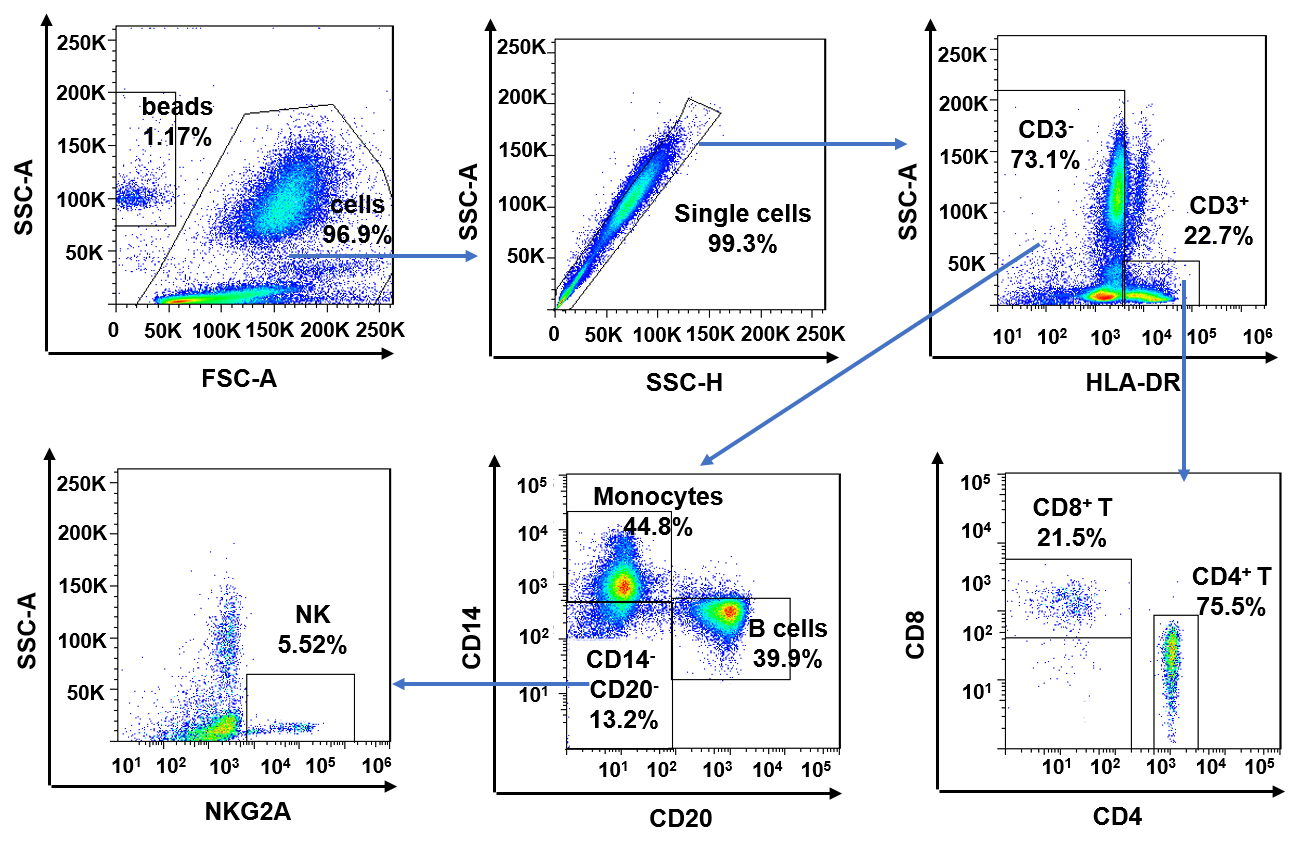
**

**Fig. S2.** Gating strategy of flow cytometry for CD4^+^ T cell, CD8^+^ T cell, B cell, NK cell, and monocyte counting.


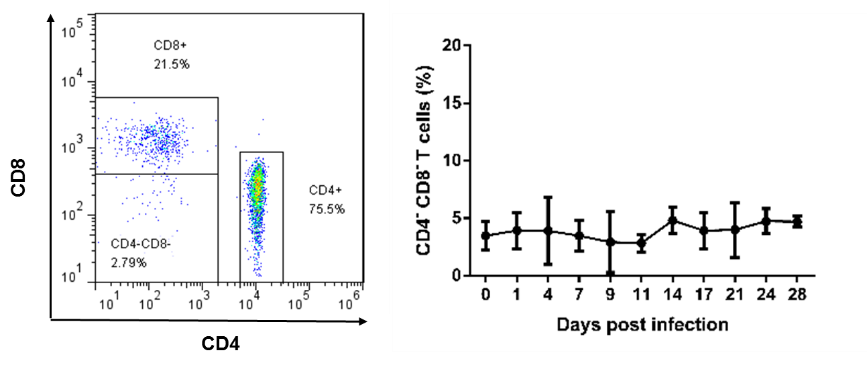


**Fig. S3.** The percentage of CD4^-^CD8^-^ T cells to T cells before and after infection.

**
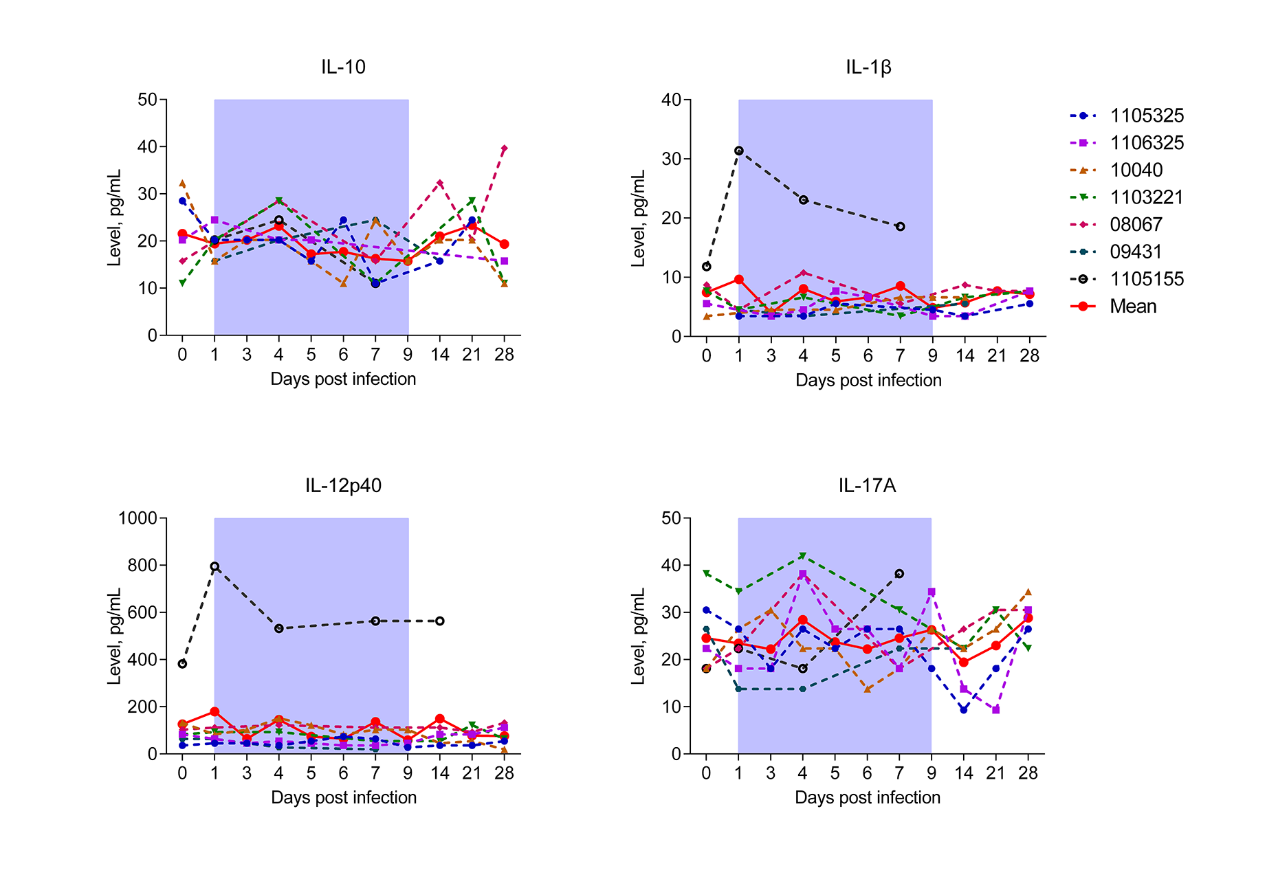
**

**Fig. S4.** Kinetics of proinflammatory cytokines in SFTSV-infected rhesus macaques.

**Supplementary Table 1**. The data of blood routine, blood biochemistry, coagulation and bsolute counts of each leukocyte population and their specific subsets, as well as their proportions.
